# Sleep and Avoidant Restrictive Food Intake Disorder (ARFID): Correlation With Psychopathology, Gender, and Academic Performance

**DOI:** 10.7759/cureus.25628

**Published:** 2022-06-03

**Authors:** Fauzia Mahr, Grace Brennan, Marley Billman, Susan Lane-Loney

**Affiliations:** 1 Pediatrics/Eating Disorders, Penn State College of Medicine, Hershey, USA; 2 Psychology, Yale University, New Haven, USA; 3 Adolescent Medicine/Eating Disorders, Penn State College of Medicine, Hershey, USA

**Keywords:** gender comparison, internalizing symptoms, cognitive performance, arfid, sleep

## Abstract

Objective

No studies have investigated sleep disturbance in avoidant/restrictive food intake disorder (ARFID). We examined sleep disturbance in ARFID and its association with eating problems, body mass index (BMI), gender, internalizing and externalizing symptoms, cognitive performance, and academic difficulties.

Methods

Data from 71 ARFID patients from our partial hospitalization program (PHP) for children and adolescents were examined. Sleep data were extracted from measures administered at admission including Achenbach Child Behavior Checklist (CBCL), Children's Depression Inventory (CDI), and Revised Children's Manifest Anxiety Scale (RCMAS). Correlational analyses were conducted to evaluate the convergent validity of parent-reported and participant-reported sleep problems. Association with the severity of eating problems, BMI, percentage of median body weight (% MBW), age, gender, psychotropic medication, psychopathology, and academic difficulties was examined using analysis of variance (ANOVA) and Pearson’s correlation.

Results

Fifty-two percent of parents and 74% of participants reported two or more sleep symptoms. Trouble sleeping was reported by 46.48% and nightmares by 35.21% of parents. Parent-reported trouble sleeping highly correlated with internalizing disorders. Parent-reported trouble sleeping and participant-reported difficulty sleeping positively correlated with attention and attention-deficit/hyperactivity disorder (ADHD) problems. Parent-reported less sleep and feeling tired correlated with sluggish cognitive tempo, while walking/talking in sleep negatively correlated with school performance. Gender differences were noted in parent-reported sleep problems. Sleep disturbances were not associated with lower BMI or median body weight at intake. Parent-reported talking/walking in sleep and participant-reported bad dreams and bedtime worries positively correlated with Children's Eating Attitudes Test (ChEAT) scores at intake and discharge.

Discussion

Our results provide compelling evidence to screen for sleep disturbance in ARFID patients regardless of median body weight and BMI. Exploration of sleep disturbances in ARFID using objective measures is warranted.

## Introduction

Avoidant Restrictive Food Intake Disorder (ARFID) is characterized by restrictive eating not driven by body image or weight concerns, leading to nutritional deficiencies, dependence on supplemental feedings, and/or psychosocial impairment. Sensory sensitivity, fear of negative consequences, or lack of interest/appetite commonly underlie the restrictive behaviors. ARFID has a heterogeneous presentation and leads to a multitude of nutritional deficiencies, medical complications, and psychiatric sequelae [1]. The prevalence of ARFID is fairly high and ranges from 0.3% to 24.6% in non-clinical and clinical populations respectively [2].

Patients with ARFID tend to experience chronic symptoms, are younger, and experience comorbid psychiatric illnesses particularly anxiety more frequently compared to other eating disorders [3,4]. Out of the many comorbid issues accompanying restrictive eating, sleep problems have received little attention. While about 19-30% of children in the general population experience problems with falling or staying asleep, more than 50% of patients struggling with eating disorders experience sleep disturbance [5,6]. The hypothalamic nuclei regulating sleep and appetite are proximally located and are possibly implicated in sleep disturbance in eating disorders [7]. Additionally, levels of appetite suppressant (leptin) and appetite stimulant (ghrelin) are sensitive to sleep duration [8]. These findings seem to establish a possible association between sleep and nutritional status. Sleep disturbance has been associated with adverse psychological and medical outcomes [9,10].

To our knowledge, no studies have investigated sleep disturbance in patients with ARFID nor its association with psychiatric comorbidities, academic performance, or cognitive performance. In view of this gap, we sought to investigate these questions in the well-characterized sample of ARFID patients in eating disorders child and adolescent Partial Hospitalization Program (PHP) from a tertiary care academic center. Thus, the aims of this study were to 1) characterize sleep disturbance in ARFID patients, 2) examine the association of sleep disturbance with BMI and % median body weight (MBW) in ARFID, 3) examine the association of sleep disturbance with internalizing and externalizing behavior problems, and 4) examine the association of sleep disturbance with sluggish cognitive tempo and school performance in ARFID.

## Materials and methods

Participants

**Figure 1:**
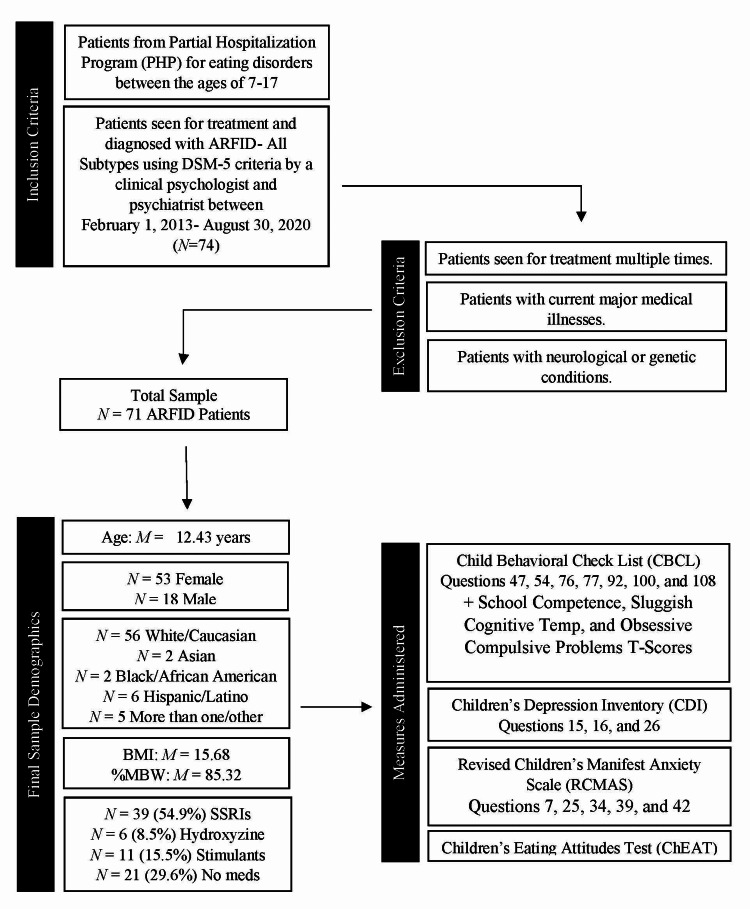
Flow chart of the screening process for selecting patients in the retrospective chart review. DSM-5 = Diagnostic and Statistical Manual of Mental Disorders, 5th Edition, BMI = Body Mass Index, MBW = Median Body Weight, SSRI = Selective Serotonin Reuptake Inhibitor, ARFID = Avoidant/restrictive Food Intake Disorder

The participants were 71 children and adolescents diagnosed with ARFID enrolled in our PHP for eating disorders between February 2013 and August 2020 ranging in age from 7 to 18 years of age and included 53 females and 18 males (Figure 1). This study was approved by the Institutional Review Board of the Penn State College of Medicine (#8749). A score of 1 or higher on sleep-related questions on the following validated measures was considered a report of sleep disturbance. Parent and self-report measures were administered on their first day of admission to the PHP with the exception of the Children’s Eating Attitudes Test (ChEAT) which was administered at admission and discharge. BMI and % MBW was also calculated on the first day of admission to PHP using the Centers for Disease Control and Prevention (CDC) guidelines.

Measures

*Achenbach Child Behavior Checklist (CBCL)* [11]: The CBCL is a validated parent-report measure that correlates with different childhood psychopathologies. The measure assesses many sleep-related complaints and has been used in longitudinal and twin studies examining sleep [12]. Sleep questions from this assessment device include items 47 (nightmares), 54 (overtired without good reason), 76 (sleeps less than most kids), 77 (sleeps more than most kids), 92 (talks or walks in sleep), 100 (trouble sleeping), and 108 (wets the bed).

*Children’s Depression Inventory (CDI)-Second Edition *[13]: The CDI-2 is a 28-item self-report measure designed to assess depressive symptoms in children ages 7 to 17 years. Sleep items from this measure include items 15 (2 = I have trouble, sleeping every night, 1 = I have trouble sleeping many nights, and 0 = I sleep pretty well), 16 (2 = I am tired all the time, 1 = I am tired many days, 0 = I am tired once in a while), and 26 (2 = I fall asleep during the day all the time, 1 = I fall asleep during the day many times, and 0 = I almost never fall asleep during the day).

*Revised Children’s Manifest Anxiety Scale (RCMAS)-Second Edition* [14]: The RCMAS-2 is a 48-item self-report measure of anxiety symptoms designed for children ages 6 to 19 years. Sleep items included from this self-report measure included items 7 (wake up scared sometimes), 25 (it’s hard for me to get to sleep at night), 34 (I am tired a lot), 39 (I have bad dreams), and 42 (I worry when I go to bed at night). RCMAS sleep scale score range was 0 for no sleep problems reported to a maximum of 5 if all problems were reported.

*The Children’s Eating Attitude Test (ChEAT) *[15]: The ChEAT is a 26-item self-report measure that evaluates problematic eating attitudes and behaviors in children between the ages of 8-14 years old. These behaviors include dieting, purging, and oral control. Items are scored on a 6-point Likert scale, and a sum score is calculated. Higher scores on this measure indicate greater impairment.

*Body Mass Index (BMI) and Percentage of Median Body Weight (%MBW):* BMI was calculated using patient height and weight taken by clinic staff at intake. The following formula was used: BMI = weight (kg)/height (m) [16]. The percentage of MBW was calculated for age and gender by clinic staff using age appropriate CDC charts. All participants were below the age of 18 years, so % MBW was used to more accurately represent body weight.

Analyses

Data were analyzed using IBM SPSS (IBM Corp, Armonk, USA) [17]. Pearson’s correlation analyses were preliminarily used to evaluate the convergent validity of parent (CBCL) and child (RCMAS and CDI) reported sleep questions. Then, one-way analysis of variance (ANOVA) and Chi-square tests were used to examine the prevalence of sleep disturbances and differential responses between groups. Pearson’s correlations were then used to investigate correlations between demographic factors, various forms of psychopathology, cognitive and academic factors (i.e., sluggish cognitive tempo, academic performance), and sleep disturbances. 

## Results

Convergent validity of parent and child reported sleep disturbance measures

The parent-report items of sleep disturbance displayed significant positive correlations with child-report sleep-related items (with the exception of one child-report item - worrying at bedtime, which is possibly secondary to the subjective emotional experience of the participant). Parents’ reports of youth sleep disturbances largely aligned with their children’s reports of their own sleep disturbances (Table 1).

**Table 1:** Pearson r values of correlations between parent and child reported sleep items. *p < .05, **p < .01, (P) = Parent Report, (PA) = Participant Report, NM = Nightmares, DTS = Daytime Sleepiness, Tired (PA)_1_ = CDI 16,  Tired (PA)_2_ = RCMAS 34, RCMAS = Revised Children’s Manifest Anxiety Scale, CDI = Children’s Depression Inventory

Item	Trouble sleep (PA)	Tired (PA)_1_	DTS (PA)	Wakes scared (PA)	Diff. sleeping at night (PA)	Tired (PA)_2_	Bad Dreams (PA)	Bedtime worries (PA)
NM (P)	0.404^**^	0.225	0.229	0.344^**^	0.259^*^	0.249^*^	0.371^**^	0.225
Tired (P)	0.046	0.384^**^	0.275^*^	0.170	0.085	0.428^**^	0.290^*^	0.188
Less sleep (P)	0.561^**^	0.230	0.321^*^	0.214	0.289^*^	0.088	0.048	0.106
More sleep (P)	0.027	0.089	0.231	0.008	0.101	0.179	0.110	0.005
Talk/Walk (P)	0.122	0.275^*^	0.239	-0.086	0.086	0.258^*^	0.280^*^	0.040
Trouble Sleep (P)	0.468^**^	0.325^**^	0.122	0.196	0.469^**^	0.178	0.260^*^	0.202
Wets bed (P)	-0.111	-0.057	-0.043	-0.184	-0.085	-0.113	-0.200	-0.107

Prevalence of sleep disturbances among ARFID patients

Among parents of youth with ARFID, 73.24% reported the presence of at least one sleep disturbance (i.e., scored as a 1 or 2 on the CBCL) in their child. Over 50% of parents reported that their child experienced two or more sleep disturbances. Overall, the most commonly reported sleep disturbances were trouble sleeping (46%), nightmares (35%), and sleeping less than other children (31%). All of these items were reported to occur at least some of the time (Table 2).

**Table 2:** Frequency of endorsement of sleep disturbance items from the Child Behavior Checklist, Children’s Depression Inventory, and Revised Children’s Manifest Anxiety Scale Frequency counts based on an item score of 1 or 2. CBCL = Child Behavior Checklist, CDI = Children’s Depression Inventory, RCMAS = Revised Children’s Manifest Anxiety Scale

Sleep Disturbance	CBCL #	# of parents endorsing item	% of parents endorsing item
Trouble sleeping	100	33	46.48%
Nightmares	47	25	35.21%
Sleeps less than most kids	76	22	30.99%
Overtired without good reason	54	21	29.58%
Sleeps more than most kids	77	13	18.31%
Talks/walks in sleep	92	11	15.49%
Wets the bed	108	3	4.23%
Any sleep disturbance		52	73.24%
Two or more sleep disturbances		37	52.11%
Sleep Disturbance	CDI #	# of children endorsing item	% of children endorsing item
Trouble sleeping	15	35	50.00%
Tired	16	36	50.70%
Fall asleep during day	26	14	26.90%
Sleep Disturbance	RCMAS #	# of children endorsing item	% of children endorsing item
Wake up scared	7	33	46.50%
Hard to get to sleep at night	25	33	46.50%
Tired a lot	34	38	53.50%
Have bad dreams	39	36	50.70%
Worry when going to bed	42	37	52.10%
Any sleep disturbance		59	83.10%
Two or more sleep disturbances		53	74.65%

Avoidant/restrictive food intake disorder participants self-reported similarly elevated levels of sleep disturbance, with 83% of patients reporting at least one sleep disturbance (i.e., scored as a 1 or 2 on the CDI or a 1 on the RCMAS). A large percentage (75%) reported two or more sleep disturbances. The most commonly reported sleep disturbances were being tired a lot (53%), worrying when going to bed (52%), and having bad dreams (51%).

Interestingly, while around 30% of parents reported "overtired without a good reason," over 50% of ARFID participants reported feeling tired.

Associations between sleep disturbances and demographic factors

Patient Age

Patient age was significantly positively correlated with parent report of feeling overtired without good reason (r (69) = .251, p = .035) and participant report of being tired often (r (69) = .273, p = .021), and falling asleep during the day (r (69) = .460, p = .001). Older children, through self-report and parent-report, are endorsing greater levels of sleep disturbances than younger children. Age was not significantly correlated with any other parent or child-report sleep disturbance items (Table 3).

**Table 3:** Pearson r values of correlations and Chi-square values for parent and child-reported sleep disturbance items and eating disorder symptomatology, cognitive tempo, school performance, and other demographic variables. *p < .05, **p < .01, (P) = Parent Report, (PA) = Participant Report, NM = Nightmares, DTS = Daytime Sleepiness, Tired (PA)_1_ = CDI 16,  Tired (PA)_2_ = RCMAS 34, BMI = Body Mass Index, MBW = Median Body Weight, ChEAT = Children’s Eating Attitudes Test Total Score, CDI = Children’s Depression Inventory, RCMAS = Revised Children’s Manifest Anxiety Scale, ADHD = Attention-Deficit/hyperactivity Disorder

Item	NM (P)	Tired (P)	Less sleep (P)	More sleep (P)	Talk/Walk (P)	Trouble. Sleep (P)	Wets bed (P)	Trouble sleep (PA)	Tired (PA)_1_	DTS (PA)	Wakes scared (PA)	Diff. sleeping at night (PA)	Tired (PA)_2_	Bad Dreams (PA)	Bed time worries (PA)
Age	-0.057	0.251^*^	0.051	0.080	-0.061	-0.014	0.096	0.030	0.273^*^	0.460^**^	0.096	-0.133	0.117	-0.142	0.042
Sex	6.71^*^	6.43^*^	2.24	0.090	1.10	2.15	6.24^*^	4.56	3.03	2.11	3.65	1.50	1.90	1.20	1.90
BMI	0.033	0.139	0.011	0.237^*^	0.166	0.076	0.047	0.129	0.311^**^	0.358^**^	0.062	-0.109	0.088	0.017	-0.104
%MBW	0.103	-0.056	-0.140	0.197	0.205	-0.036	-0.006	0.009	0.118	-0.080	0.054	-0.120	-0.053	-0.001	-0.047
ChEAT Intake	0.173	0.180	0.058	0.132	0.282^*^	0.188	-0.115	0.101	0.173	0.130	0.159	0.090	0.163	0.238^*^	0.273^*^
ChEAT Discharge	0.043	0.139	0.117	0.106	0.410^**^	0.019	-0.033	0.181	-0.068	0.247	0.144	0.143	0.033	0.228	-0.056
Attention Problems	0.253^*^	0.094	0.445^**^	-0.041	0.264^*^	0.387^**^	0.040	0.213	0.188	0.130	0.013	0.237^*^	0.119	0.108	0.203
ADHD Problems	0.262^*^	0.153	0.309^**^	0.031	0.222	0.322^**^	-0.007	0.169	0.203	0.151	0.010	0.253^*^	0.105	0.114	0.146
Sluggish Cognitive Tempo	0.142	0.285^*^	0.318^**^	0.022	0.117	0.196	0.053	0.078	0.179	0.127	0.056	0.099	0.192	-0.048	0.120
School Performance	-0.205	-0.078	-0.126	-0.154	-0.286^*^	-0.176	0.002	-0.079	-0.065	0.022	-0.111	-0.118	-0.141	-0.097	-0.128

Patient Gender

There were significant differences between responses of parents of male and female patients on the following sleep disturbance items: nightmares (X2 (2, N=70) = 6.7, p=.035), overtired without good reason (X2 (2, N=70) = 6.4, p=.040), and wets the bed (X2 (2, N=70) = 6.2, p=.044) (Table 3). Parents of female patients reported higher severity of nightmares and overtiredness, while parents of male patients reported higher severity of bed-wetting. No significant differences between male and female patients' self-report sleep disturbance items emerged.

Internalizing and externalizing symptoms

Parent-reported externalizing behaviors on the CBCL (comprising Rule-Breaking Behavior and Aggressive Behavior scales) were positively correlated with the following child-reported sleep disturbances: being tired a lot (r (69) = .390, p = .001), having bad dreams (r (69) = .394, p = .001), and worrying when going to bed (r (69) = .378, p = .001). Parent-reported internalizing behaviors on the CBCL (comprising Anxious/Depressed, Withdrawn/Depressed, and Somatic Complaints scales) were positively correlated with all of these items as well as waking up scared (r (69) = .271, p = .022).

Specific forms of psychopathology

Overall, sleep disturbance (parent-reported CBCL item “trouble sleeping”) positively correlated with the following CBCL psychopathology scales: anxious/depressed, somatic complaints, thought problems, attention problems, affective problems, anxiety problems, and ADHD problems.

Parent report of sleep disturbance was not significantly correlated with self-reported anxiety (total RCMAS scores) or depression (total CDI scores) however, participant-reported sleep disturbance (CDI self-report item 'trouble sleeping') positively correlated with somatic complaints and thought problems (Table 4). 

**Table 4:** Pearson r values of correlations between sleep disturbance items and behavioral concerns. *p < .05, **p < .01, (P) = Parent Report, (PA) = Participant Report, NM = Nightmares, DTS = Daytime Sleepiness, Tired (PA)_1_ = CDI 16,  Tired (PA)_2_ = RCMAS 34, CDI = Children’s Depression Inventory, RCMAS = Revised Children’s Manifest Anxiety Scale

Item	Trouble Sleep (P)	Trouble sleep (PA)	Tired (PA)_1_	DTS (PA)	Wakes scared (PA)	Diff. sleeping at night (PA)	Tired (PA)_2_	Bad Dreams (PA)	Bed time worries (PA)
Internalizing	0.327^**^	0.217	0.302^*^	0.166	0.271^*^	0.166	0.390^**^	0.394^**^	0.378^**^
Externalizing	0.212	0.064	0.250^*^	0.223	0.171	0.090	0.313^**^	0.268^*^	0.286^*^
Anxious/ Depressed	0.353^**^	0.154	0.348^**^	0.051	0.295^*^	0.156	0.423^**^	0.425^**^	0.433^**^
Somatic Complaints	0.287^*^	0.258^*^	0.276*	0.307^*^	0.189	0.249^*^	0.349^**^	0.366^**^	0.248^*^
Thought Problems	0.588^**^	0.395^**^	0.384^**^	0.228	0.302^*^	0.364^**^	0.382^**^	0.297^*^	0.382^**^
Affective Problems	0.533^**^	0.225	0.433^**^	0.231	0.236^*^	0.189	0.458^**^	0.355^**^	0.313^**^
Anxiety Problems	0.296^*^	0.129	0.211	-0.003	0.266^*^	0.154	0.277^*^	0.292^*^	0.419^**^

Associations between sleep disturbances and sluggish cognitive tempo/academic difficulties

The CBCL sluggish cognitive tempo scale (comprised of four items: “confused or seems to be in a fog,” “daydreams,” “stares blankly,” and “lacks energy/underactive”) was positively correlated with parent-reported overtiredness without good reason (r (69) = .285, p = .016) and sleeping less than most kids (r (69) = .318, p = .007), suggesting an association between sleep disturbance and cognitive difficulties. Furthermore, talking or walking in sleep was negatively correlated with the school performance scale (comprised of four items, “mean score of performance in academic subjects,” “special education or remedial services,” “repeated grade,” or “any academic problems in school”) (r (69) = -.286, p = .018), suggesting an association between parasomnias and academic difficulties (Table 3). Sluggish cognitive tempo and school performance were not significantly correlated with any participant-reported sleep disturbances.

Sleep disturbance and BMI/median body weight

Sleep disturbance was not associated with lower percent median body weight. The following sleep disturbance items were positively correlated with BMI at intake: sleeping more than most kids during the day (r (69) = .237, p = .047), being tired often (r (69) = .311, p = .008), and falling asleep during the day (r (69) = .358, p = .009) (Table 3). 

Sleep disturbance and severity of eating disorder pathology (ChEAT scores)

The Children’s Eating Attitude Test scores at intake were positively correlated with parent-reported walking and talking in sleep (r (69) = .282, p = .018). ChEAT scores also positively correlated with participant-reported bad dreams (r (69) = .238, p = .047) and worrying when going to bed (r (69) = .273, p = .022). Scores at discharge were also positively correlated with parent-reported walking and talking in sleep (r (69) = .410, p = .001). Our results indicate that the severity of eating pathology may be associated with specific sleep disturbances in ARFID. (Table 3)

Sleep disturbance and medication effect

Participants were divided into four groups (SSRIs (Selective Serotonin Reuptake Inhibitors), hydroxyzine, stimulants, and no meds) based on the medications taken at intake (Figure 1). These groups were compared on their responses to parent- and child-reported sleep disturbances. No significant differences were found.

## Discussion

This is the first study examining sleep disturbances in patients with ARFID. Eighty-three percent of ARFID participants reported at least one symptom of sleep disturbance, while their parent reports indicated at least one symptom of sleep disturbance in 73.24% of ARFID participants. The parent-reported measures of sleep disturbance displayed positive correlations with each of the child-report sleep-related items with the exception of “worrying at bedtime,” which is possibly secondary to the subjective emotional experience of the participants. The positive correlation strengthens our findings and confirms that ARFID patients experience significant sleep disturbances.

Over 50% of parents reported that their child experienced two or more sleep disturbances and 75% of ARFID patients indicated the presence of two or more symptoms of sleep disturbance. Overall, the most commonly reported sleep disturbances were trouble sleeping, nightmares, and sleeping less than other children, all reported to occur at least some of the time. These results provide compelling evidence to screen for sleep in ARFID.

Patient age was significantly correlated with patient feeling overtired without good reason and with feeling tired often, and falling asleep during the day per participant reports but did not significantly correlate with any other parent or child-report sleep disturbance items. Insufficient sleep plays a central role in feeling tired [18]. Additionally, irregular sleep patterns during teenage years may contribute to daytime sleepiness and feeling tired. Objective determination of sleep duration can help clarify the correlation between developmental age and tiredness.

Even though there were no significant differences between male and female self-reported sleep disturbance, there were significant differences between parental reports of male and female participants regarding the following sleep disturbance items: nightmares, overtired without good reason, and wets the bed. Parents of female patients reported higher severity of nightmares and overtiredness, while parents of male patients reported higher severity of bed wetting. These findings confirm the previous findings in the literature regarding the higher prevalence of enuresis in males [19]. 

Gender difference with higher sleep disturbances in girls has been previously reported [5]. Daytime tiredness has been commonly reported during puberty and has been attributed to hormonal changes and irregular sleep-wake cycles [18]. The mean age for patients in our ARFID cohort was 12.43 years, which coincides with the onset of puberty and may account for the gender difference observed in our sample. Previous studies conclude that the risk for depression doubles with puberty and correlates with insomnia symptoms at that developmental stage in girls [20]. While the effects of malnutrition cannot be overlooked, the possible impact of reproductive hormones on sleep disturbances requires attention.

Sleep disturbance was strongly associated with internalizing symptoms (anxious/depressed). These findings confirm previous literature on adolescents which reports that adolescents experience more internalizing symptoms, and the severity of anxiety in adolescence correlates with a higher number of sleep problems [16,21]. Another study also found that sleep problems were associated with both internalizing and externalizing behaviors [4]. Our results also demonstrated that somatic complaints, and affective and thought problems strongly correlated with sleep disturbances. ARFID patients experience higher anxiety compared to other eating disorders, which may also account for these findings in association with sleep disturbance experienced by ARFID participants [3,4]. 

Sluggish cognitive tempo positively correlated with parent-reported overtiredness without good reason and sleeping less than most kids, suggesting an association between sleep disturbance and cognitive difficulties. Furthermore, talking or walking in sleep was negatively correlated with school performance, suggesting an association between parasomnias and academic difficulties. There is evidence that cognitive processes are heavily influenced by sleep quality, and sleep disturbances may adversely affect attention, learning, and memory [22,23]. Mounting evidence suggests a positive association between sleep loss and poor academic performance [24]. Disrupted sleep has long been associated with attention and learning difficulties [9]. Our results confirm these findings of attention problems with sleep disturbance. Notably, management of sleep disorders improves cognitive functioning [25,26]. These findings underscore the importance of sleep management in ARFID patients presenting with academic difficulties. Sluggish cognitive tempo in ARFID warrants longitudinal follow-up to assess possible mitigating effects of nutritional rehabilitation.

Sleep disturbances were not associated with low BMI or a low percentage of median body weight at admission. This finding may be secondary to the fact that many ARFID patients experience nutritional deficiencies which are not always reflected in BMI or MBW. Furthermore, nutritional restriction in ARFID does not always lead to weight loss [27]. Therefore, exploration of nutritional deficiencies beyond BMI or MBW must be explored to identify the role of specific nutritional deficits underlying sleep disturbances in ARFID.

The ChEAT scores reflected a strong correlation of sleep disturbance with dieting (restrictive eating) at admission. This finding substantiates the above results and findings imply that restrictive eating/dieting may lead to specific sleep disturbance in ARFID. Indeed tryptophan, a precursor of serotonin, decreases with restricted eating, and its supplementation has shown to improve sleep quality [28].

There were no significant differences in group responses regarding reports of sleep disturbance between participants taking various psychotropic medications. In contrast to existing literature, we did not find any differences in parent or participant sleep reports between the med vs no med groups. Current literature suggests that there are differences between subjective reports and objective measurements of sleep disturbances in patients taking psychotropic medication [29]. Future studies should focus on the objective measurement of sleep in ARFID to clarify these findings.

Several limitations should be considered when reviewing our study. First, this is a retrospective cross-sectional study, so conclusions regarding causality cannot be drawn. Second, the reported sleep problems are based primarily on the subjective reports of parents and participants. Despite the strong item-level correlations between parent and child reports which provides evidence for the validity of these measures, we acknowledge that the lack of objective sleep measurements is a limitation. However, CBCL item “trouble sleeping” has been reported to strongly correlate with sleep latency assessed by sleep diary and longer sleep latency assessed using actigraphy [12]. Third, we relied on parents to provide information about cognitive and academic performance. Collateral information from school reports would have substantiated our findings. The reports of feeling tired could have been substantiated by exploring sleep duration and bedtime practice, and by excluding the presence of behavioral insomnia. Many participants in our cohort were taking psychotropic medication and their effect on sleep cannot be ruled out even though we found no group differences in sleep disturbance between different medication groups or between medication and no medication group.

## Conclusions

This is the first study reporting sleep disturbance in ARFID and provides novel insights regarding behavioral correlates, gender effects, BMI, eating pathology, and academic performance. Our findings emphasize the importance of screening for sleep disturbance in ARFID regardless of weight and BMI status. Future longitudinal studies should explore objective sleep disturbance in ARFID using PSG (polysomnography) or actigraphy. The role of specific nutritional deficiencies, bio-regulatory systems, environmental factors affecting sleep including parenting practices, sleep hygiene, association with treatment response, and the degree to which nutritional recovery may ameliorate sleep disturbance in ARFID warrant further investigation.
